# CNS Diseases and Uveitis

**Published:** 2011-10

**Authors:** Pia Allegri, Roberto Rissotto, Carl P. Herbort, Ugo Murialdo

**Affiliations:** 1 Uveitis Center, Ophthalmology Department, Rapallo Hospital, Genova, Italy; 2 Neurology Department, San Martino Hospital, Genova, Italy; 3 Center for Ophthalmic Specialized Care, Lausanne, Switzerland; 4 University of Lausanne, Lausanne, Switzerland

**Keywords:** CNS Inflammatory and Infectious Diseases, Eye Inflammatory and Infectious Diseases, Uveitis

## Abstract

A number of inflammatory, infectious, neoplastic and idiopathic disorders affect the eye and the central nervous system (CNS) concurrently or at different time frames. These conditions pose a diagnostic challenge to the clinician since they may present with similar ocular and neurological manifestations. The purpose of this review is to describe major neurological syndromes including multiple sclerosis, Vogt-Koyanagi-Harada disease, other autoimmune syndromes, and several infectious diseases which may affect the eye. This article may serve as a guide for the diagnosis and treatment of such disorders. It should be noted that these conditions have been viewed from a neurologist’s perspective thereby neurologic involvement is stressed.

## INTRODUCTION

The frequent concurrence of uveitis and central nervous system (CNS) diseases may be derived from the common embryogenic developmental pathway of the posterior segment and the CNS. Analogies between the blood brain barrier and the blood retinal barrier, and “immune privilege” involved in the prevention and blockage of noxious immune system reactions both in the eye and CNS also reflect the common origin of these organs. A North-American review^1^ revealed that about 8% of individuals suffering from uveitis have associated neurological symptoms, however “neurological” patients seldom have uveitis, probably because neurologists usually consider the CNS disease as the leading condition requiring treatment.

Many CNS disorders affect the posterior segment of the eye. Despite their heterogeneous origin (demyelinating, infectious, inflammatory-autoimmune and neoplastic), upon which the most recent classification is based, these diseases have a single functional unit as their target, represented by the CNS and the eye.

Such pathogenetic associations may underlie variability in prevalence related to ethnicity. For instance Behçet disease, which is rare in the USA (0.3% of uveitic entities)^1^, is very frequent in the Turkish population (more than 30%)^2^,^3^.

Apart from these considerations, we also have to point out that, the most represented patients in Smith’s work was a group of people suffering from CNS disease of unknown origin (meningitis and myelitis) or peripheral nervous system (PNS) disease (angiitis).^1^

From an etiological point of view, the two broadest and most recent surveys from the USA (1,450 patients)^1^ and Europe (Austria, 1,973 subjects)^4^, are not univocal regarding the etiology of conditions described above. The American analysis shows that about 8% (115/1,450 subjects) with neurological diseases had associated uveitis among which demyelinating disorders, Vogt-Koyanagi-Harada (VKH) disease and lymphomas were more frequent. On the other hand, the European study showed that only 2.6% (52/1,973 patients) had a neurological/uveitis related disease, the most prevalent of which were demyelinating, VKH, bacterial, Behçet’s and lymphomatous disease.

This review will discuss major entities which present with neurological and ocular inflammatory diseases.

## CLASSIFICATION

The following classification is a modification based on the work by Brazis et al.^5^

Demyelinating diseases

- Multiple sclerosis
- Monosymptomatic optic neuritis

Infectious diseases

- Viral (herpes virus, cytomegalovirus, West Nilevirus, human T-cell lymphotropic virus (HTLV-1), acquired immunodeficiency syndrome (AIDS), subacute sclerosing panencephalitis (SSPE)
- Bacterial (cat-scratch disease, Whipple’s disease)
- Mycobacterial (tuberculosis, leprosy)
- Spirochetal (lyme, syphilis)
- Protozoal (toxoplasmosis, pneumocystis carinii)
- Nematodal or parasitic (gnathostomiasis)

Inflammatory or Autoimmune

- Polyarteritis nodosa
- Wegener granulomatosis
- Rheumatoid arthritis and Sjögren’s syndrome
- Systemic lupus erythematosus
- Sarcoidosis
- Behçet’s disease
- VKH disease
- APMPPE (acute posterior multifocal placoid pigment epitheliopathy)

Neoplastic

- Lymphoma
- Paraneoplastic syndromes

## DEMYELINATING DISEASES

Multiple sclerosis (MS) may affect the entire CNS and must be distinguished from monosymptomatic optic neuritis (MSON) which presents with ocular symptoms but no systemic involvement. MSON can cause a single episode, or lead to multiple recurrences of ocular involvement. A number of patients, after some years of quiescence, evolve to MS such that 40% of MSON cases develop MS in 8 to 10 years (Fig. 1A).

Isolated or combined with other neurological symptoms, optic neuritis mainly affects young patients (less than 30 years of age) and is rarely seen after the age of 45. In the latter case, the disease presents with medullary deficits and a rapidly disabling course. Based on such multiform clinical findings, one may assume that MS is not a single clinical entity, but a group of demyelinating diseases which can be differentiated from each other not only on the basis of symptoms and course, but also according to etiology, genetics, immunopathogensis and response to therapy.

Optic neuritis usually presents with acute unilateral blurring of vision, transient photopsia, ocular or orbital pain (due to optic nerve edema) and other neurological complaints including sensory, motor, cerebellar and urinary bladder symptoms. Other symptoms such as diplopia, vertigo (due to proximity of the oculomotor area to the equilibrium area), trigeminal neuralgia, neuro-vegetative deficits (mainly orthostatic hypotension) and cognitive deficits are infrequent.

The course of MS is characterized by relapses and remissions (more commonly during the early stages of the disease) but may occur as a primary or secondary chronic-progressive disease. In later stages, a number of variations and mixed forms such as chronic-progressive disease with overlapping relapses are possible.

Magnetic resonance imaging (MRI) shows hyperintense round/oval demyelinating lesions mainly in periventricular areas on T2-weighted sequences (Fig. 1B). If the same lesions appear hypointense on T1-sequences, axonal damage may accompany the demyelinating lesion (Figures 2A and 2B).

A classic acute attack of MS or MSON usually lasts more than 24 hours; if visual or non-visual symptoms are transient, they represent only brief deterioration due to CNS causes leading to nerve conduction delay. In these cases symptoms rapidly disappear when the exciting cause subsides.

Intermediate uveitis is associated with MS; 10–20% of uveitic subjects show MS symptoms after some years, and a similar number of MS cases present with major anatomo-pathological signs of intermediate uveitis. These may include retinal peri-vasculitis mainly affecting veins in the active phase, vascular sheathing in the chronic phase, and perivenous sclerosis following retinal vasculitis in over 30% of chronic-progressive MS patients (Fig. 1A, left frames). Retinal vein inflammation is directly related to activity of the systemic disease and portends an unfavourable prognosis. This typical association between retinal vasculitis and MS finds its anatomical explanation in the common origin of the blood brain and blood retinal barriers, and their inflammatory breakdown.

Another inflammatory ocular association of MS is recurrent granulomatous or non-granulomatous acute anterior uveitis which usually presents bilaterally but asymmetrically.

The genotypic prevalence of MS associated-uveitis was recently investigated in a study which found a high predominance of HLA-DR_15_ and DR_2_ genotypes.6 HLA associations identify subjects at risk of developing the disease but they are not diagnostic markers. With the exception of age, no significant differences have been observed in patients with and without associated MS.

## INFECTIOUS DISEASES

### Herpes Virus Infections

Herpetic infections, including herpes simplex virus (HSV) types 1 and 2, varicella zoster virus (VZV), cytomegalovirus (CMV), and Ebstein-Barr virus (EBV), are diseases that typically affect immunocompromised, HIV-positive, atopic, or transplanted individuals or patients receiving cytostatic, steroid or immune-suppressive therapy. However, an increasing incidence is noted nowadays in healthy people. The status of the host’s immune system defines the outcome of these infections and complications related to these viral entities.

Ocular involvement presents in two major forms: 1) anterior uveitis and 2) viral retinopathies.^8^ Viral retinal involvement is subdivided into different clinical entities, namely: necrotizing retinopathy including acute retinal necrosis (ARN) and progressive outer retinal necrosis (PORN), and non-necrotizing herpetic retinopathy (NNHR), that affects subjects under corticosteroid therapy for autoimmune diseases such as Behçet’s disease.

ARN is usually caused by VZV and HSV, but more rarely by CMV^9^,^10^ and is typically unilateral. It affects both healthy and immunocompromised patients and is characterized by acute necrotizing retinitis with retinal arteriolitis, optic neuritis and severe vitritis.^7^ PORN is frequently bilateral, affects immunocompromised subjects and is characterized by mild anterior uveitis without vitritis and an acute necrotizing retinitis beginning in the posterior pole extending toward the retinal periphery.^11^ NNHR presents as bilateral chronic and atypical posterior uveitis.^12^

All herpetic infections show tropism for sensory ganglia inside which the virus remains dormant during periods of latency. This mechanism explains how herpes viruses spread to ganglia-derived areas (dermatomes).

Neurological manifestations are usually not related to immune system dysregulation or suppression. HSV neurological infections cause: 1) encephalitis and 2) recurrent aseptic meningitis.

Encephalitis is mainly related to reinfection or reactivation of HSV type 1 in immunocompetent subjects; HSV type 2 primary infections affect immunocompromised patients and re-infections are rare (4–6%). Necrotizing acute encephalitis is frequently localized to the temporal lobes, sometimes with meningeal involvement, and presents with typical symptoms of meningoencephalitis (high fever, headache, sensory abnormalities), associated with memory, personality and speech disturbances because of hippocampal**,** frontal and temporal lobe lesions respectively (Fig. 2C).^13^,^14^

VZV infection typically presents with sensory ganglia involvement including thoracic or cranial (mainly ophthalmic division of trigeminal nerve) ganglia with dermatomal cutaneous manifestations. In the central nervous system, VZV produces herpes zoster ophthalmicus which can involve the eye where it produces anterior granulomatous uveitis often resulting in sectorial iris atrophy. Although herpes zoster is due to reactivation of dormant varicella virus, it is an acute viral infection that requires prompt treatment with systemic antivirals to avoid complications which occurred in the era before antivirals were available.^15^ VZV encephalitis and other viral CNS inflammations are less frequent than HSV infections.

Apart from these classical forms, a new neurological entity called “Zoster sine herpete” has been described, thanks to the recent introduction of polymerase chain reaction (PCR) tests. It presents as aseptic meningitis or encephalitis (with cerebellar tropism), sometimes as transverse myelitis, or cerebral vasculopathy derived from granulomatous angiitis (late CNS vasculitis) with deep ischemia of the basal ganglia, temporal lobes, and the internal capsule.^9^

Primary herpetic infection results in axoplasmic transport of the virus to sensory ganglia where it establishes latency. Reactivation leads to retrograde virus transport. The true path by which the virus reaches the CNS has not been established, one possibility is that the reactivated virus located in ganglia may spread to the anterior and middle cranial fossa.

The mechanism by which herpes viruses affect the eye is the double occurrence of a lytic reaction followed by intraocular inflammation.^12^

History and clinical examination of herpetic infections of the CNS and eye are usually diagnostic because they are well-defined entities. In controversial or atypical presentations, molecular diagnosis of cerebrospinal or ocular fluids by PCR provides high sensitivity (96%) and specificity (99%)^10^,^12^ for detection of herpetic DNA.

For diagnostic purposes in equivocal cases of meningoencephalitis, the following techniques may be useful: 1) cerebrospinal fluid (CSF) analysis revealing typical features of monocytic pleocytosis, high protein content and normal glucose content; 2) neuro-imaging studies including computed tomography (CT) or ideally MRI with characteristic findings of symmetric necrosis of the temporal lobes; 3) electroencephalography typically showing periodic spike waves on temporal derivations. These investigations may facilitate diagnosis leading to prompt and specific therapy with systemic antiviral agents, preventing severe ocular and neurological sequelae and sometimes death.^13^

Recurrent aseptic meningitis is mainly due to HSV 2; history is positive for herpes genitalis and this infection seems to spread via the oculogenital route to the eye and via sacral sensory ganglia to the CNS. It presents with symptomatic meningitis and fever, meningism, an intense headache lasting for some days followed by alternating recovery with symptomatic periods. Typical CSF findings are lymphocytic pleocytosis and high protein levels. PCR investigation is almost always positive; viral culture is positive only in the early stages of the disease.

CNS and ocular infections due to CMV have been reported following congenital infections and in patients with AIDS in the pre-HAART (highly active anti-retroviral therapy) era due to severe immunodeficiency, sometimes causing death. Currently, the course of CMV retinitis or meningoencephalitis in immunocompromised hosts has dramatically changed and CMV infection may present as the first and only clinical manifestation of AIDS.

CMV infection in the eye presents as active retinitis with a typical “pizza-like” appearance consisting of white-yellowish cotton wool areas of necrotizing retinitis associated with hemorrhage and vascular sheathing with mild vitritis evolving towards progression of retinitis and retinal scarring. Disk atrophy follows papillitis or acute anterior optic neuritis in the acute phase 16.

CMV encephalic infection is mainly localized to the brainstem (similar to Behçet’s disease). Typical symptoms are lid ptosis, oculomotor disturbances and nystagmus. Ventriculitis and inflammatory poly-neuropathies are infrequent.

Bell’s palsy may be caused by any of the viral diseases under this category; however, to date, there has been no association with uveitic entities.

### Human T-cell Lymphotropic Virus Type 1

HTLV-1 is the cause of T-cell leukemia and of a peculiar myelopathy (tropical spastic paraparesis) which may be associated with ocular manifestations including keratoconjunctivitis sicca, corneal lesions, retinal vasculitis and uveitis, otherwise known as HTLV-1 associated uveitis or HAU. It is more frequent in Japanese, African, Caribbean and South-American patients.^17^ Transmission occurs through sexual contact, needle injection or sharing, and also vertically from mother to child.

## AIDS

Indirect AIDS consequences are opportunistic infections and tumors. HIV-1 viral infection can directly induce a number of neurological syndromes^18^ which can present as an acute disease (aseptic acute meningitis and meningoencephalitis) or as a chronic disorder (HIV encephalopathy, AIDS-dementia complex). These conditions are difficult to diagnose because of the very slow progression of cognitive and behavioral impairment which can be misdiagnosed as reactive neuropsychological symptoms. MRI usually shows cortical and subcortical atrophy and T_2_ hyperintense areas related to demyelinization.

Ocular manifestations of AIDS can be categorized into four main groups^19^: micro-angiopathy manifesting as cotton wool spots and vascular occlusions; opportunistic infections including CMV, herpetic, toxoplasmic, fungal and parasitic retinitis; neoplasms such as lymphomas and skin carcinomas particularly Kaposi sarcoma; and neuro-ophthalmic presentations including papilledema, papillitis, visual field defects and oculomotor palsies.

### West Nile Virus

West Nile virus (WNV) infection is a mosquito-borne disease widely distributed in all continents. Since 1999, when the first outbreak of WNV encephalitis occurred in New York city, it has had an impressive spread, representing a recent example of geographical evolution of a disease formerly restricted to specific areas.^20^

WNV, similar to other arbovirus (flavivirus family) transmitted infections such as dengue, yellow fever and Japanese encephalitis, shares the common feature of neuro-invasiveness with grey matter affinity, with loci of involvement in the cortex, basal ganglia, brainstem, cerebellum and spinal cord. Less than 1% of infected individuals develop severe neuro-invasive diseases which can be classified into three major but sometimes overlapping, clinical syndromes: meningitis, encephalitis and acute flaccid paralysis.^21^

Meningitis has the usual manifestations: fever, headache and neck stiffness. Encephalitis shows focal symptoms related to the site of involvement such as epilepsy (cortex), tremor and involuntary movements (basal ganglia), parkinsonism (substantia nigra) and ataxia (cerebellum). Encephalitis incurs severe sequelae and a high incidence of death. Acute asymmetric flaccid paralysis, similar to poliomyelitis (a selective lesion in anterior spinal horns), has an unfavourable outcome with variable recovery.

Before the onset of ocular or CNS symptoms, many patients are healthy without any signs of immunosuppression, while some (approximately 20%) complain of mild flu-like symptoms including headache, fever, malaise, gastrointestinal upset, skin rash, neck rigidity and changes in mental status.

Ocular manifestations are uncommon although chorioretinitis may be more frequent than previously reported.^22^ Bilateral multifocal chorioretinitis with typical clinical and fluorescein angiographic features including linear clusters of oval chorioretinal lesions primarily located in the posterior pole or in the mid-periphery along major retinal vessels, is the most common finding followed by other ophthalmic manifestations such as anterior uveitis, vitritis, retinal vasculitis, and optic nerve involvement (optic neuritis and optic disc swelling).

Detection of WNV–specific IgM in the serum or CSF provides strong evidence of recent infection. IgM usually becomes detectable 8 days after the onset but may become elevated after some months^22^. Therefore, WNV-IgM may reflect previous infection rather than recent disease. Direct detection of WNV in the CSF, serum or tissue specimens by viral isolation is also of possible diagnostic value. Patients with neuro-invasive disease show lymphocytic pleocytosis in the CSF. MRI is usually normal, but signal modifications may be seen in the basal ganglia, thalamus, brainstem and anterior spinal cord of patients with encephalitis or acute flaccid paralysis.

### Subacute Sclerosing Panencephalitis

Subacute sclerosing panencephalitis (SSPE) is an encephalopathy of subacute course affecting children and adolescents caused by an aberrant measles virus called the SSPE virus. It is characterized by progressive encephalopathy involving both grey and white matters with a fatal outcome in most cases.^23^

Ocular involvement is seen in more than half of the cases and is usually bilateral. The macular area is usually involved by retinitis or pigmentary changes. Optic neuritis is present in two-thirds of cases with ocular involvement. Ocular findings may sometimes precede CNS signs and symptoms. Macular involvement may resemble acute posterior multifocal placoid pigment epitheliopathy (APMPPE) which is one of the differential diagnoses.^24^

### Syphilis

Ocular involvement in subjects affected with syphilis is strongly suggestive of CNS involvement and should be considered synonymous with neuro-syphilis.^25^ Neuro-syphilis has protean clinical patterns^26^ including early or secondary syphilis (asymptomatic neurological, meningeal and meningovascular involvement), and late or tertiary syphilis (progressive general paralysis and tabes dorsalis). Antibiotic treatment reduced the prevalence of the disease in the years following World War II but nowadays, a rising incidence is being observed due to immigration waves and changes in sexual habits.

From an ophthalmological point of view,^27^ secondary syphilis may cause: 1) cranial mono-neuropathies, i.e. oculomotor nerve palsies, due to vasa nervorum vasculitis, meningeal inflammatory reaction, intracranial pressure increase or gumma production; 2) optic neuritis and perineuritis related to meningitis; 3) Argyll-Robertson pupil due to lamina quadrigemina infectious involvement.

Acute meningitis can occur in 1 to 2% of patients with secondary syphilis, and can cause papilledema due to a rise in intracranial pressure. With papillitis and vitritis, patients complain of acute visual loss; neuroretinitis manifests with hard exudates in the peripapillary area. Another manifestation is optic “perineuritis” which may be suspected in a subject with optic nerve head swelling without ocular symptoms which is due to mild inflammation of meningeal sheaths of the optic nerve. Many patients with ocular signs do not show systemic evidence of the syphilitic disease.

Ocular manifestations also include anterior (granulomatous and non-granulomatous) uveitis, intermediate uveitis, panuveitis, chorioretinitis, retinal vasculitis, and necrotizing retinitis; the latter may be the first presentation in patients with HIV infection. Ocular syphilis has to be treated exactly in the same way as neuro-syphilis.

Tertiary syphilis may present with meningovascular and late parenchymal consequences and from an ophthalmological point of view, with interstitial keratitis or different forms of uveitis. The slower and more chronic course of tertiary syphilis is the only difference between secondary and tertiary stages of the disease. A small number (5%) of tertiary neuro-syphilis patients suffer from optic atrophy.

The protean expressions of syphilis, particularly ocular involvement, have caused the disease to be designated as “the great imitator”.

### Lyme Disease

Lyme disease is a tick-borne infection due to different types of Borreliae, called “the new great imitator” since it displays a broad variety of systemic symptoms ranging from skin erythema migrans to neurologic or cardiac lesions. Ocular manifestations^28^ usually appear in late stages of the disease. Early manifestations of the disease may include transient conjunctivitis and episcleritis; they may reappear in late stages which are typically characterized by interstitial keratitis, uveitis (anterior, intermediate, posterior and panuveitis), choroiditis and retinal vasculitis.

**Figure 3:**
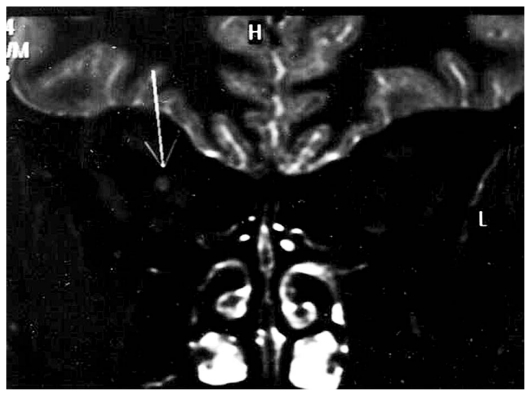
Lyme disease: MRI appearance of right optic nerve enhancement after administration of paramagnetic contrast (T1-weighted image).

Neuro-ophthalmic manifestations^29^, in contrast to ocular signs, appear at an earlier stage (in the second or dissemination phase). Borreliae pass the blood brain barrier in the first weeks of the infection, and may lead to symptoms of cranial and spinal nerve involvement (with facial nerve neuropathy being the most common neuropathy, comprising 50% of all neurologic symptoms of Lyme disease). Optic nerve involvement (neuritis, papilledema, neuroretinitis) can also occur during this stage (Fig. 3).

Cranial neuropathies may result from direct infection, autoimmune inflammatory response (i.e. vasculitis of vasa nervorum), or raised intracranial pressure. Trigeminal nerve involvement may present as bilateral decrease in corneal sensation or as ocular and periocular pain. Papilledema may occur with meningitis and increased intracranial pressure. Horner’s syndrome, tonic or dilated pupils, and photophobia have also been described as signs of encephalopathy or meningitis.

In the third stage, circumscribed vasculitic brain lesions appear and result in different symptoms according to the site of involvement (dementia, ataxia and spastic paraparesis) and an MRI pattern similar to that of MS (multiple hyperintense white matter lesions on T_2_ weighted images). In demyelinating disease, involvement of basal ganglia grey matter is absent.^29^,^30^

Serological tests for Borrelia lack adequate sensitivity and specificity, but PCR may be a useful diagnostic tool. Early antibiotic therapy is the mainstay of management; steroids can be added in subjects with severe intraocular or encephalic inflammation.

### Toxoplasmosis

**Figure 4 (A and B):**
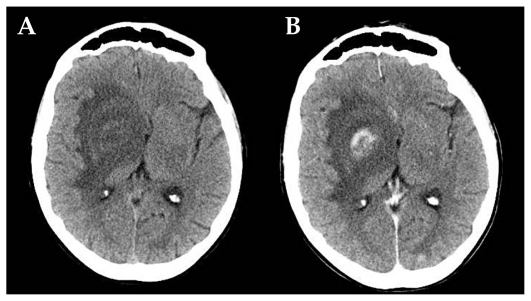
AIDS-related neuro-toxoplasmosis: CT scan of a toxoplasmic localization in the right capsulonuclear region with wide peri-lesional edema before (A) and after (B) contrast injection.

Neuro-toxoplasmosis is the major cause of encephalitis and retinitis in immuno-compromised hosts occurring in 1–3% of patients. One third of AIDS patients suffer from this disease (Figures 4A, 4B, 5A and 5B). Papilledema is also seen in affected eyes.

From a neurological point of view, lesions may be localized to the basal ganglia, causing symptoms such as involuntary movements^31^ or in cerebral hemispheres with a predilection for the occipital lobes producing typical visual field defects. Fever, headache, seizures, and sensory deficits are accompanying features of these symptoms.

Major ocular lesions^32^ in immuno- compromised patients are foci of necrotizing retinitis presenting as large bilateral multifocal areas of retinitis which are also typical of congenital infection (Fig. 5C).

Immunocompetent individuals show small and isolated lesions of necrotizing retinitis without CNS involvement. These acute lesions appear as white foci of retinitis with poorly defined margins healing as a pigmented and atrophic scar. Chorioretinal toxoplasmic lesions are self-limiting only in immunocompetent individuals.

Diagnosis is mainly clinical although serological investigations including PCR and local antibody production detection are helpful in atypical cases.

In immunocompetent patients, anti-toxoplasmic therapy is only useful in terms of reducing the rate of recurrence, but does not seem to be effective in the acute stage. Such therapy, in vision threatening disease, may be strengthened by systemic steroid administration which is useful in controlling the inflammatory reaction and accelerating recovery. Anti-toxoplasmic antibiotic therapy is useful in immunosuppressed patients, but corticosteroids are not required in severely immunocompromised patients.

### Whipple’s Disease

Whipple’s disease (WD) is a multi-systemic infection with predominantly gastrointestinal manifestations caused by an actinomyces (Tropheryma Whippelii). Major presenting symptoms include diarrhea, impaired intestinal absorption and progressive weight loss. Once the organism enters the bloodstream via the intestinal lamina propria, ‘colonization’ of the CNS occurs in a variable number of cases (43–100%) according to differing studies. Asymptomatic phases alternate with sudden relapses, suggesting direct action of the microorganism^27^. Neurologic symptoms are due to involvement of cerebral hemispheres (dementia, myoclonus, and epilepsy), hypothalamus (insomnia, hyperphagia, polydipsia) and brainstem (nystagmus, supra-nuclear ophthalmoplegia, diplopia and lid ptosis). The rare but pathognomonic sign is ophthalmo-masticatory myorhythmia, which consists of abnormalities of vertical eye movements with pendular nystagmus associated with tongue and mandibular movements. The most common symptoms are dementia, which may be reversible, followed by supranuclear ophthalmoplegia, myoclonus and hypothalamic disorders.

Ocular manifestations are secondary to CNS involvement. According to a large review^33^,^34^, WD ocular involvement appears to be present in about 4% of all cases, with a variety of manifestations including anterior uveitis, vitritis, retinal vasculitis, chorio-retinitis, optic disc edema and optic atrophy.

### Cat Scratch Disease

Cat scratch disease is a systemic zoonosis caused by a gram-negative bacillus (Bartonella henselae). Systemic infection after primary inoculation is associated with localized erythema and regional lymphadenitis with flu-like symptoms.

The major neurological manifestation (in 1–2% of cases) is encephalopathy from infective micro-localizations, involving both hemispheres and underlying cerebral structures. Characteristic symptoms are epileptic seizures^29^ and personality disorders (especially aggressiveness) due to cerebral lesions, and lethargy caused by brainstem localization. Less frequent signs are cranial (especially facial) neuropathies.

The largest reported case series^35^ included 76 patients, 61 with encephalopathy and 15 with cranial neuropathies.

When the eye is involved, the disease is characterized by Parinaud’s oculoglandular syndrome including red eye, mucoid conjunctival exudates and unilateral lymphadenopathy. After hematogenous spread neuroretinitis, chorioretinal lesions (typically juxtavascular), and sometimes capillary angiomatous proliferation may be noted.^35^,^37^

### Gnathostomiasis

Gnathostomiasis is a parasitosis due to nematodes of the genus *Gnathostoma* (mostly G. spinigerum, sometimes G. hispidum), which is endemic in subtropical Asia (from Thailand to Japan) and in some parts of the American continent (especially in Mexico). In the “visceral” stage of its complicated life cycle which follows the “cutaneous” stage, this parasite can involve all internal organs, including the eye and CNS. Neurological manifestations are associated with a high percentage of mortality and include (in order of appearance): radiculo-myelitis, radiculo-myelo-encephalitis, eosinophilic meningitis and subarachnoidal hemorrhage (SAH). Parasites enter the CNS through nerve roots (both spinal and cranial) and give rise to symptoms (e.g. radicular pain), which persist for some days. Afterwards, limb paralysis (from a single to all four limbs, mostly paraplegia) can occur when parasites ascend to the brain. Finally, eosinophilic meningitis and SAH, the most frequent causes of death, ensue. In Thailand, 6% of all SAH in adults and 18% of SAH in children appear to be due to gnathostomiasis.

The eye is the only organ where gnathostoma can be visualized, usually in the anterior chamber, but also rarely in the vitreous. Ocular symptoms include uveitis (mostly anterior), iritis, intraocular hemorrhage, glaucoma, retinal scarring and retinal detachment.

Because of the “immune privilege” status of the eye, eosinophilia is less marked with ocular disease than when other organs are involved.^38^

## OTHER INFECTIOUS DISEASES

Anterior uveitis can occur in meningococcal sepsis, a potentially fatal illness and less frequently with meningococcal meningitis.

The most frequent neurological manifestation of tuberculosis is meningitis, which unlike other causes of bacterial meningitis is characterized by clear CSF with monocytic pleocytosis due to rupture of sub-ependymal tubercles. Intra-parenchymal granulomas can induce epilepsy. Other symptoms of neuro-tuberculosis are papilledema, retrobulbar optic neuritis and oculomotor nerve palsy. Ocular involvement is polymorphic and any form of uveitis is possible, the most common being posterior uveitis, serpiginous-like choroiditis and subretinal abscesses due to foci of caseous necrosis.

Mycoplasma pneumoniae infection can cause aseptic meningitis. Symptoms related to involvement of the left hemisphere, i.e. right hemiparesis and motor aphasia, have been described by Perez and Foster^15^ as well as sixth and seventh cranial nerve palsy. The eye may be affected with mild anterior uveitis.

Neurological involvement in cryptococcosis includes meningitis (presenting with nausea, drowsiness, irritability, headache and neck stiffness), peri-optic meningeal infiltration leading to retrobulbar optic neuritis and cranial nerve (mostly sixth nerve) palsy, and parenchymal localization causing dementia. In 40% of cases of neuro-cryptococcosis, ocular involvement is present, mostly as multifocal chorioretinitis.^18^ Leptospirosis has an initial phase of blood diffusion and a second “immune phase” due to circulating antibodies, which in 50% of cases includes aseptic meningitis with monocytic pleocytosis and cranial nerve palsies. At the same time, acute anterior uveitis with fine keratic precipitates and posterior synechiae can occur. Cataract development is also a frequent complication.

Leprosy affects the peripheral nervous system causing mainly sensory poly-neuropathy with neuropathic pain and paresthesia in the first stage, and sensory loss with susceptibility to traumatic limb injuries in more advanced stages. Acute or chronic anterior uveitis may be present in 10–50% of cases.^18^,^19^

## OCULO-CEREBRAL VASCULITIS

Cerebral vasculitides (CVs) are associated with retinal vasculitis and uveitis in different percentages. CVs are present in a number of systemic autoimmune disorders and show common clinical aspects: 1) a symptomatic pattern including headaches (due to stimulation of perivasal nervous endings), multifocal deficits (secondary to ischemic lesions) and neuropsychiatric symptoms (due to ischemic lesions or, as in SLE, related to auto-antibodies); 2) blood signs of acute inflammation; 3) coexistence of central and peripheral neurological deficits (e.g. mononeuritis multiplex, similar to vasa nervorum vasculitis); 4) frequent extra-neurological involvement, because vasculitides are systemic diseases.

**Figure 6:**
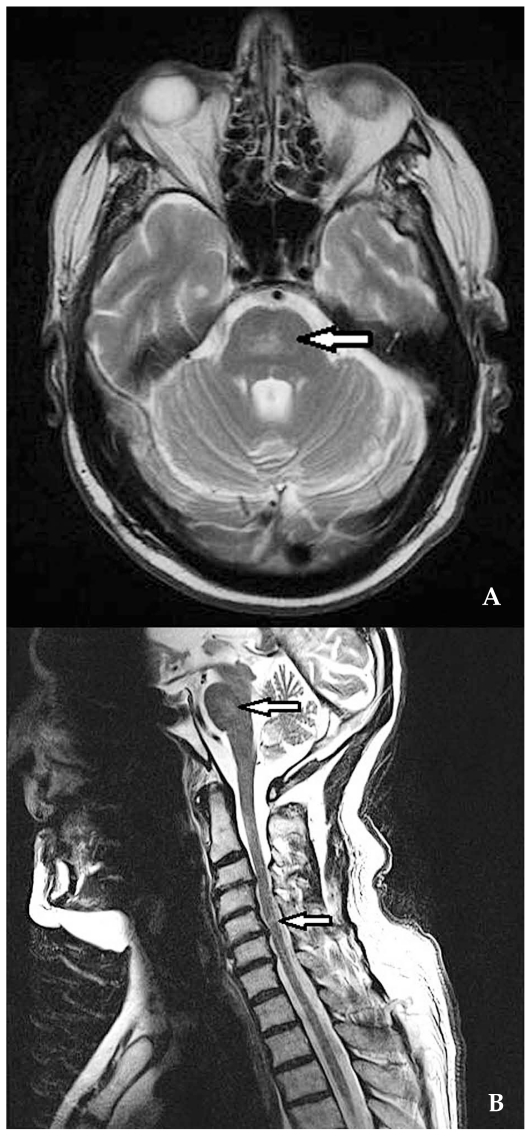
MRI in disseminated CNS vasculitis (T2-hyperintense lesions) with localizations, in the same patient, both in the central pontine area (A) and in the cervical cord (B).

On MRI vasculitic lesions are usually hyperintense on T2- but sometimes also on T1-weighted sequences (Figures 6A and 6B). If an isolated CV presents, CSF analysis is essential but for a definite diagnosis, a cerebral biopsy may be necessary.

CVs which commonly affect the eye include polyarteritis nodosa, Wegener’s granulomatosis, rheumatoid arthritis (more often, Sjögren’s syndrome) and systemic lupus erythematosus.

Polyarteritis nodosa (PAN) is a necrotizing segmental inflammation of small and middle sized arteries; it is frequently (70%) associated with hepatitis B virus (HBV) seropositivity and clinically presents as peripheral involvement (mononeuritis multiplex in 70% of cases) as compared to CNS lesions (ischemic or hemorrhagic stroke in 10%). Vasculitic involvement can affect all ocular structures leading to episcleritis, diffuse/nodular scleritis, vasculitic or occlusive retinopathy, choroidal vasculitis (the most frequent manifestation), ischemic optic neuropathy and orbital vasculitis (with extraocular muscle ischemia and amaurosis fugax).^39^

**Figure 7:**
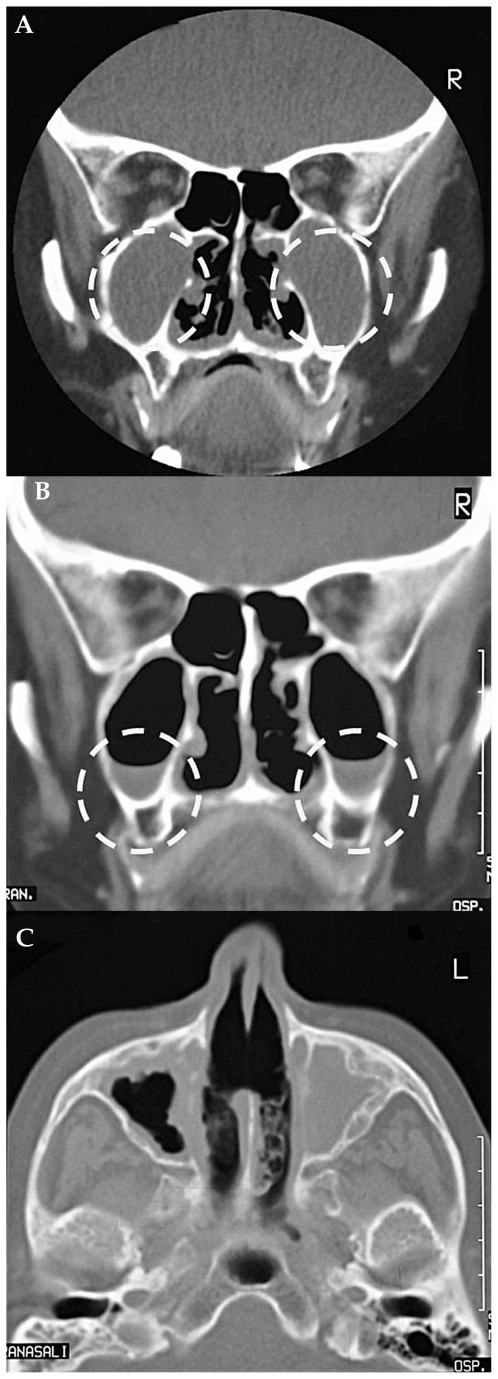
Wegener’s granulomatosis (CT scan without contrast): the maxillary sinuses are filled with granulomatous tissue **(A)**; the same lesions, after treatment, show reduction in granulomatous invasion **(B)**; perforation of the nasal septum **(C)**.

Wegener’s granulomatosis (WG), together with Churg-Strauss arteritis and microscopic polyangiitis, is one of the ANCA (anti-neutrophil cytoplasmic antibody)-positive vasculitides. Neurological manifestations are caused by invasion from paranasal sinuses to the cranial nerves causing oculomotor nerve deficits^40^, to the hypothalamus leading to diabetes insipidus, and to the meninges with aseptic meningitis (Figures 7A–C). Angiitis with lymphoreticular infiltrates and necrosis can produce direct vascular lesions: e.g. ischemic optic neuropathy, or ischemic cerebral lesions with focal symptoms (strokes, seizures). As with all systemic diseases, WG can also affect all ocular structures including the choroid, but with a predilection (in contrast to PAN) for the anterior segment and orbit.

Rheumatoid arthritis (RA) can be the cause of central or, more frequently peripheral (vasa nervorum) vasculitic involvement; however, retinal vasculitis is rare. Sjögren’s syndrome, an independent or RA-linked disease which was once thought to be a variant of RA, presents with frequent, both central and peripheral neurological symptoms which precede the diagnosis of the condition in most cases.^41^ Ischemic CNS lesions, both supra-spinal and spinal, are present.^42^ Supra-spinal lesions cause optic neuritis (in more than 20% of cases with CNS involvement), cognitive impairment, and epilepsy. Spinal lesions produce acute or chronic myelopathy. Sjögren’s syndrome mimics the clinical course of MS at the central level and in the neuro-radiological pattern. Peripheral involvement generates any type of neuropathy due to vasa nervorum vasculitis.^42^ The most common and typical form is a predominantly sensory polyneuropathy. Autonomic neuropathy, with orthostatic hypotension and Adie’s pupil, is less frequent. Biopsy shows a typical pattern of axonal neuropathy (as a rule in ischemic neuropathies), with axonal loss, angiitis and perivascular infiltrates.

**Figure 8:**
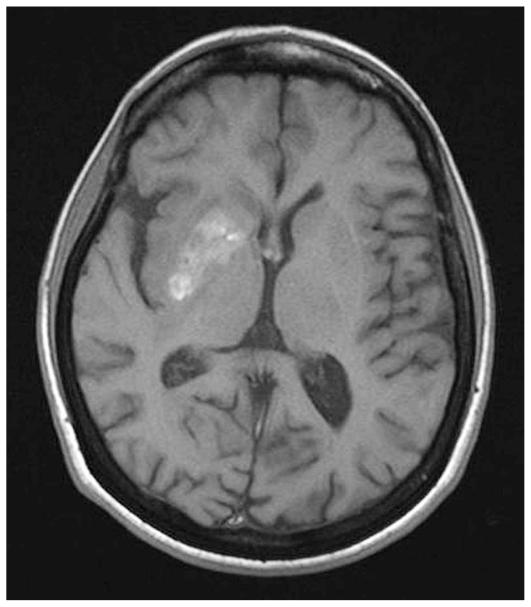
CNS vasculitis in lupus: spontaneous T1-hyperintense lesions in the right striatum.

Systemic lupus erythematosus (SLE), one of the most polymorphous diseases, can cause neurological manifestations with different mechanisms. Focal symptoms (epileptic seizures, motor and sensory deficits, extra-pyramidal symptoms) are due to circumscribed lesions of vasculitic nature (Fig. 8). At onset, thrombotic vasculopathy, immune complex vasculitis, anti-phospholipid antibody syndrome, or embolization from Libman-Sacks endocarditis can be present. SLE has an apparent tropism for the vertebrobasilar system, with a high frequency of brainstem symptoms (transient diplopia, vertigo, internuclear ophthalmoplegia) and for the occipital lobes (visual hallucinations, hemianopsia, geniculo-calcarine blindness). Also, headaches frequently have a vasculitic origin.

Neuropsychiatric disorders (depression, psychosis) are caused by direct action of anti-neuron antibodies including anti-P (anti-ribosomal P-protein) and anti-N (anti-neuronal cell) antibodies.^43^

As in PAN and WG, any ocular structure can be affected, from the lacrimal system to the retina. Inflammatory arteriolar vasculopathy is more severe in the presence of anti-cardiolipin antibodies and is often complicated by proliferative retinopathy in the case of major vascular occlusion, by pigmentary alterations (“pseudoretinitis pigmentosa”), or by exudative retinal detachment (secondary to choroiditis) although less common than retinal involvement.^44^

## SARCOIDOSIS

Sarcoidosis is a protean multisystemic disease of unknown origin caused by dysfunctional T-lymphocytes, phagocytes, and epithelioid cells generating non-caseating granulomatous multi-organ inflammation. Neurological and ocular manifestations of sarcoidosis are uncommon and present in 5 to 10% of cases according to different reports. No predisposing factors are evident. Neuro-sarcoidosis (NS) begins in the leptomeninges and spreads through the subarachnoid space to other meningeal structures, the hypothalamus, hypophysis, chiasma and cranial nerves, and through perivascular areas towards encephalic parenchyma. Infrequently, encephalic vasculitis may be present.

Meningeal localization is evident in more than 90% of autopsies but clinical manifestations of such involvement are less frequent (8–64%).^45^ A recent neuro-imaging survey^45^ showed that only 1/3 of affected patients were MRI-gadolinium enhanced positive for meningeal involvement.

According to different studies,^45^–^48^ neurological symptoms have discordant incidences and are classified as:

- Optic neuritis (present in 8–13% of symptomatic patients) is caused by granulomatous optic nerve infiltration, intracranial hypertension following meningitis, extrinsic compression, and vasa nervorum vasculitis. Related symptoms are pupillary abnormalities (tonic pupils) and hemianopsia (occipital visual pathway involvement).
- Single or multiple cranial nerve involvement caused by nerve infiltration, vasa nervorum vasculitis, intracranial hypertension or compression. The seventh cranial nerve is mainly involved (20–50%) because of parotid compression, followed by oculomotor manifestations (diplopia), the so-called Parinaud’s syndrome.
- Peripheral nerve sensory-motor neuropathy.
- Cerebral localization (generalized seizures and psychiatric symptoms).
- Granulomatous infiltration myopathy.
- Neuroendocrine symptoms (15–23%) including hypogonadism and diabetes insipidus because of granulomatous involvement of the hypothalamus and hypophysis.

Polymorphic ocular manifestations of sarcoidosis may coexist; these include adnexal involvement (lacrimal glands, extraocular muscles, lid granulomas), conjunctivitis, episcleritis, scleritis, keratitis, granulomatous or non-granulomatous anterior uveitis, posterior uveitis (subretinal granulomas, retinal vasculitis), optic nerve involvement (granulomas of the optic disc, papillitis, papilledema). Uveitis is the most frequent sign in NS^48^ and anterior uveitis is present in 1/3 of subjects.

**Figure 9:**
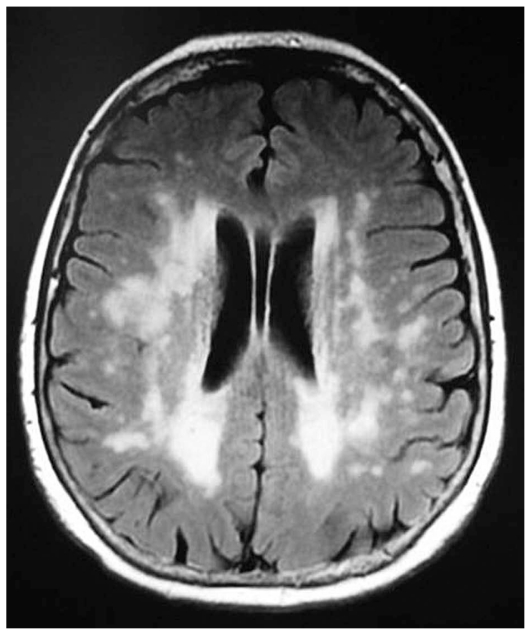
Neuro-sarcoidosis: multiple granulomas in the white matter of both brain hemispheres (contrast enhanced T1-weighted MRI image). Focal parenchymal lesions are T2-hyperintense and T1-isointense (with homogeneous contrast enhancement, as in this figure).

**Figure 10:**
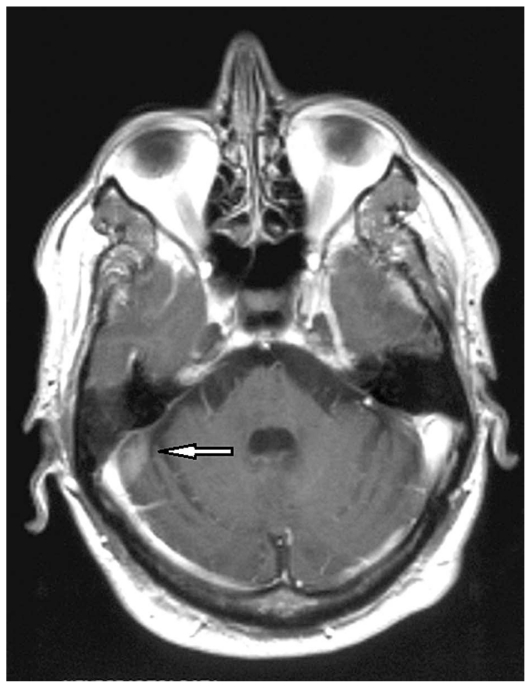
Neuro-sarcoidosis (T1 MRI sequence): homogeneous contrast enhancement of the leptomeninges and of a granuloma located in the posterior fossa.

Ocular manifestations in subjects suffering from NS are more common than previously thought. Patients with neurological symptoms and associated intraocular inflammation should undergo work-up for sarcoidosis including brain and orbital MRI (Figures 9 and 10) and lumbar puncture; tissue biopsy should be performed if there are clinically accessible lesions.

Oral steroids are the mainstay of treatment for NS and associated intraocular involvement; when they fail, systemic immunosuppressive or biological agents can also be used to avoid chronic sequelae of the disease.

## BEHCET’S DISEASE

Behçet’s Disease (BD) can be defined as a systemic vasculitis that usually affects venules, sometimes veins, rarely arterioles and exceptionally arteries. These different frequencies remain unexplained as yet. The eye is the most commonly involved organ, with typical manifestations of relapsing-remitting panuveitis and retinal vasculitis. As a systemic disease, BD may also affect the CNS.

Neuro-Behçet (N-BD) shows ethnic variations and also disparity within the same ethnic group and from one study to another; this depends on inclusion criteria and characteristics employed in each study. The following illustrates this variability. In a group of 558 Turkish BD subjects Akman-Demir^49^ detected a 29% of N-BD patients among which, 38 patients (6.8%) with angio-Behçet were found; another Turkish author, Kural-Seyahi^50^ showed that N-BD affects males (13%) more than females (5.6%); Hamdan^51^ demonstrated that among 90 Lebanese subjects, 20 (23%) suffered from it and 2 (2.2%) had angio-Behçet; Benamour^52^ found a 16.6% of N-BD subjects in a group of 925 Moroccan patients. B’chir Hamzaoui^53^ pointed out that 11.6%of cases among 519 BD Tunisian patients had CNS involvement; Borhani-Haghighi^54^ in a previous smaller epidemiological statistic, found 8.7% of N-BD patients but in a wider statistic (690 patients) found only 2.6%.

The frequency of BD neurological manifestations varies from 3% to 30% and seems to be high among Arabs, intermediate in Turkish subjects and low in Japanese individuals.^55^ Neurological or gastroenteric onset of BD is considered a negative prognostic factor. According to the type of affected vessels, N-BD displays various manifestations: 49,54,56–64

- If small veins are involved, the condition corresponds to the meningo-parenchymatous form (MP).
- If veins are affected, the term angio- BD is employed.
- If small arteries (vasa vasorum) are involved, aneurysms may rarely develop.

MP is the prevalent form and the MP/ angio-BD ratio varies from 3/1 to 11/1 according to different statistics. Male subjects are more frequently affected by BD and N-BD. The pathogenesis of MP and angio-BD is an unsolved problem; we only know that the two forms have different pathogenic mechanisms, in fact they rarely appear in the same patient.

MP shows tropism for the brainstem in which the venous circle appears to have slower blood flow than other districts; however, only the mesencephalon and pons are involved whereas the bulbus remains undamaged. Other preferred CNS structures are the cerebellum, basal ganglia, internal capsule and meninges.

The course of MP is very similar to MS, with relapsing-remitting primary and secondary chronic-progressive forms; the only difference is in the sudden onset of symptoms and the lower frequency of MP relapses. As a relapse symptom, headaches are very frequent, are mostly hemicranial (with or without aura) rather than meningeal, and are related to lesions in the periaqueductal grey matter (a pontine structure involved in the pathogenesis of migraine).

Bilateral pyramidal signs are the most common symptoms of MP; other symptoms include behavioral disorders (at onset, personality regression; later, cognitive impairment, subcortical dementia and psychiatric manifestations) and less frequently brainstem signs, e.g. several forms of ophthalmoplegia (not only the internuclear kind, as in MS). Meningeal symptoms are rare despite the specific “meningo-parenchymatous” involvement, optic neuritis is also rare.

Intracranial hypertension is the main presentation of angio-BD and is due to thrombosis of venous sinuses (sinus sagittalis superior and sinus transversus) where flow is low. Intracranial hypertension gives rise to papilledema and often abducens palsy from extrinsic compression. Headaches are very frequent in the acute phase and are sometimes associated with fever.

When compared to MP, angio-BD shows an earlier outbreak, an invariably acute course (it is never chronic-progressive), a better response to therapy and a more favorable prognosis.

Rare forms of N-BD are: aneurysms, indirectly caused by vasa vasorum vasculitis and by sectorial arterial wall ischemia (the encephalic circle is infrequently compromised but the abdominal aorta and iliac arteries are frequently affected); pseudotumor (mimicking a neoplasm on neuro-radiological investigations), characterised by bulky localisation of the MP form; different types of neuropathies (from vasa nervorum vasculitis; the presence of electrophysiologic axonal-type alterations, as in ischemic neuropathies, has been reported in patients without neuropathic symptoms^65^).

From an ophthalmological point of view^66^,^67^, the eye may show different lesions ranging from severe non-granulomatous anterior uveitis with sterile hypopyon to vitritis, ischemic necrotizing retinitis or occlusive retinal vasculitis. Fluorescein angiography (FA) is the most useful imaging modality used for assessment of inflammatory and occlusive involvement of retinal vasculature and for detection of complications such as pre-retinal neovascularization. Color Doppler

**Figure 11 (A–B):**
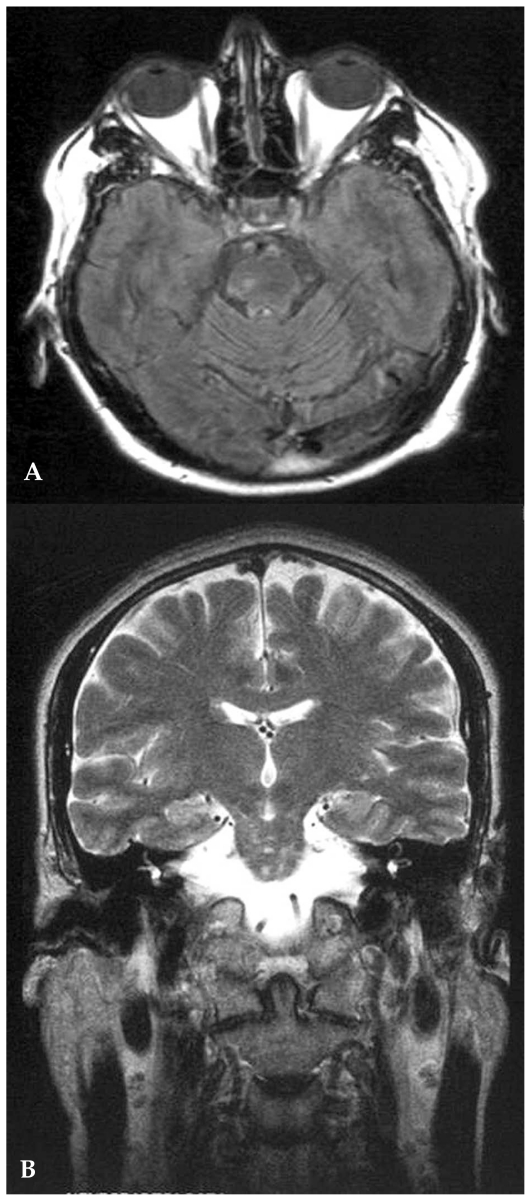
Behçet‘s disease, meningo-parenchymatous form: pontine lesions on axial **(A)** and coronal **(B)** T2-weighted MRI images. ultrasonography shows orbital and ocular hemodynamic alterations.

**Figure 12:**
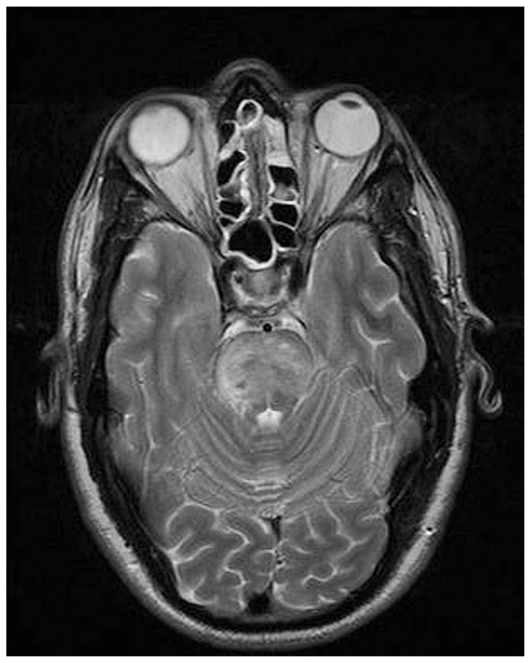
Behçet ‘s disease, meningo-parenchymatous form: wide truncal signal alterations (mostly T2-hyperintense, with some T2-hypointense foci).

Major diagnostic techniques for BD are CSF analysis which shows blood-brain-barrier damage, pleocytosis, and sometimes oligoclonal bands; MRI which shows different patterns in the MP subtype according to the stage of the disease. In early stages, the lesions are T1-isointense and T2-hypointense; in subacute and late stages, the same lesions are both T1- and T2-hyperintense (Figures 11A, 11B and 12). Flow absence corresponding to the presence of a thrombus is seen in angio-BD. VEPs are often abnormal, despite the rarity of optic neuritis, with a peculiar pattern which shows a very small amplitude in the acute stage without increased latency (unlike MS). Single photon emission computed tomography (SPECT) is positive, although many cases do not show neurological signs demonstrating early cerebral involvement.

Recently, Turkish authors reported that in patients with BD uveitis, treatment with oral cyclosporine seems to be associated with an increased risk of MP forms, although the reason is unknown.^67^

## VOGT-KOYANAGI-HARADA DISEASE

Vogt-Koyanagi-Harada (VKH) disease is one of the most common uveitic-related neurological diseases. This condition affects other apparently heterogeneous tissues including the leptomeninges, cochlea and skin which are similar in the presence of melanocytes. In fact, during embryogenesis, most pigment cells arise from cranial or truncal portion of the neural crest and migrate to the skin, hair bulbs, choroid, inner ear, leptomeninges and other tissues.^68^ VKH syndrome is an autoimmune disease which “targets” three enzymes involved in the synthesis of melanin: tyrosinase and two other related enzymes, namely tyrosinase-related protein (TRP) 1 and 2. Other target proteins also probably have a pathogenic role (MART-1, PMel-17/gp100, KU-MEL-1, PAX-3)^69^. This “overabundance” of auto-antigens supports the “antigen unmasking” hypothesis, probably triggered by a virus such as EBV, which explains direct tissue damage leading to the release and unmasking of segregated substances.

VKH is related to HLA-DR_4_ ad HLA-DR_53_ .which ;re predominant in some ethnic groups (Japanese, Chinese, North-American and Hispanic) and to the HLA-DRB 1*0405 allele, which is predominant in Brazilians 69,70.

The incidence of neuro-otologic symptoms is related to ethnicity. Meningism and the CSF pattern of monocytic meningitis are prevalent in the Japanese. Otological symptoms and perceptive deafness are more frequent in the Japanese, whereas tinnitus shows a similar distribution in all studied populations apart from Hispanic populations who are less affected by otological symptoms. Hearing involvement can persist over many years.

The meninges are affected in the prodromal stage which is characterized by fever, headache, neck stiffness and psychomotor retardation together with cochlear involvement which causes dysacusis and tinnitus. Neuro-otologic symptoms are more prominent in the second stage, when uveitis also occurs (i.e. the acute uveitic or uveitic-acoustic stage). Vestibular symptoms are rare, probably because different types of melanocytes are present in the inner ear. The skin is involved in the third stage, i.e. the convalescent stage, when “sunset glow fundus” and retinal pigmentary abnormalities can occur. In the fourth stage, if the disease is untreated, ocular complications can appear.^71^,^72^

### Ocular Manifestations

In the acute stage, fundus findings consist of bilateral multifocal serous retinal detachments in the posterior pole together with optic nerve inflammatory edema. FA reveals retinal pigment epithelium (RPE) hyperfluorescent dots, multiple round areas of dye pooling in the subretinal space, and optic disc leakage. Indocyanine green angiography (ICGA) demonstrates patchy filling delay of choroidal vessels and the choriocapillaris, diffuse dye leakage, and multiple hypofluorescent spots. Optical coherence tomography (OCT) shows large serous retinal detachments in the central area.

In the convalescent stage, fundus examination reveals the typical sunset-glow appearance with irregular linear pigmentation in the posterior pole. FA shows granular hyperfluorescence reflecting RPE damage and ICGA demonstrates significant improvement in filling delay, but hypofluorescent dark spots (choroidal granulomas) persist for a long period.^71^–^80^

### Neurologic Manifestations

In early stages, neurological manifestations appear before ocular symptoms. Main symptoms are fever, headache, nausea, photophobia, meningism and neck stiffness, orbital pain (related to optic nerve edema or hyperemia) and galea capitis hyperesthesia. A recent study identified anterior ischemic optic neuropathy (AION) with irreversible visual field defects (6 cases in 52 consecutive patients).^75^

CSF examination allows early diagnosis by showing a not very high monocytic pleocytosis (about 400/mm^3^) causing confusion with an infectious process. A recent review by Andreoli and Foster^72^ showed that CSF specimen was positive in more than 80% of cases in the first week, and in 97% of cases three weeks after the onset of symptoms. CSF changes have been reported in patients with no neurological symptoms.^76^,^77^ Lymphocytic pleocytosis, hyper-proteinorrachia, and macrophages with melanin depots are other CSF findings.

Monocytic pleocytosis can persist for a long time but usually remits after eight weeks or much later. A correlation between pleocytosis and “sunset glow fundus” in the convalescent stage was reported by Keino^77^; in fact, significant neurologic inflammation at the onset will predict the development of long term VKH complications. According to Touitou^76^, a correct diagnosis of VKH is made with the combination of international diagnostic criteria^74^ together with 1) clinical examination, 2) CSF examination (pleocytosis) and 3) FA-OCT demonstrating serous detachment in the posterior pole (present in over 98% of cases). However the most efficient tool for early diagnosis and subsequent monitoring of subclinical choroidal disease is ICGA.^78^,^79^ If choroidal disease is followed and treated using ICGA, chronicity and complications may be avoided.^80^

## ACUTE POSTERIOR MULTIFOCAL PLACOID PIGMENT EPITHELIOPATHY

**Figure 13:**
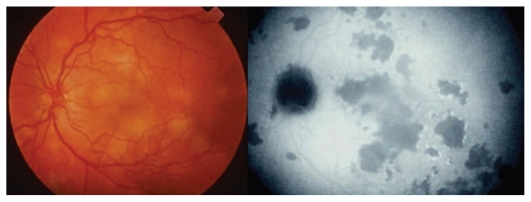
Acute posterior multifocal placoid pigment epitheliopathy (APMPPE). Multiple bilateral confluent yellowish fundus lesions corresponding to choriocapillaris nonperfusion on ICGA (right frame).

Acute posterior multifocal placoid pigment epitheliopathy (APMPPE) is a primary inflammatory choriocapillaropathy often provoked or preceded by a viral infection. The condition may present with neurological symptoms which are variable or can follow ocular manifestations. Typical ocular symptoms are photopsia, scotoma and visual loss. Ocular findings include deep bilateral areas of yellowish discoloration in the posterior pole with multiple serous exudative retinal detachments, together with mild vitritis and anterior uveitis. ICGA shows round geographic hypofluorescent areas which represent choriocapillaris non-perfusion (Fig. 13).

The most frequent neurological symptoms is meningism with lymphocytic pleocytosis and headaches.^80^,^81^ Less frequently, ischemic strokes due to cerebral vasculitis causing focal deficits, mainly involving the posterior circulation (with visual field defects) and the basal ganglia may occur. These strokes arise simultaneously or even before ocular symptoms. Another relatively rare symptom is transient hearing loss. All of these signs and symptoms lead to the suspicion that APMPPE might be a peculiar form of VKH disease.

APMPPE must be suspected in a young (20 to 30 year old) patient with a viral flu-like syndrome, stroke of unclear etiology, meningism with CSF pleocytosis, and ocular symptoms such as photopsia, scotoma and visual blurring.

## OTHER RARE INFLAMMATORY DISEASES

Kawasaki’s disease or the “mucocutaneous lymph node syndrome” can present as a bilateral anterior uveitis with or without aseptic meningitis and monocytic pleocytosis in children and young adults.

Crohn’s disease and ulcerative colitis can cause ocular signs including iritis, conjunctivitis, episcleritis, scleritis and uveitis; and inflammatory/ischemic neurological symptoms i.e. optic neuritis and other cranial nerve neuropathies, especially oculomotor nerves.

Cogan’s syndrome consists of acute interstitial keratitis and vestibulo-auditory dysfunction. The latter deficit may also occur in Eale’s disease, which is very frequent in India; the main sign is retinal periphlebitis without true uveitis.

Relapsing polychondritis is an autoimmune disease targeting type II collagen characterized by destructive and relapsing inflammation of the cartilage in organs such as the nose, earlobe and trachea. Ocular manifestations include episcleritis, scleritis and iridocyclitis; neurological symptoms are encephalic (confusional states, epilepsy and stroke). Cranial nerves, chiefly the optic nerve and sensorineural deafness, are involved in necrotizing vasculitis 15.

Susac’s syndrome (retino-cochleo-cerebro vasculopathy) is a very rare immune-mediated microangiopathy characterized by encephalopathy, branch retinal arterial occlusion (BRAO) and hearing loss of unknown etiology. The main presenting symptoms are CNS features.^82^

Presenting symptoms of encephalitis are personality and behavioral changes, associated with intense headaches and migraine. MRI shows small multifocal supra-tentorial lesions (especially in the corpus callosum) mainly involving the white matter, however, the grey matter is sometimes affected as well.

Visual symptoms are related to the site of occlusion: if in the retinal periphery (and also in encephalopathic or otherwise uncooperative patients) there are no visual complaints; if it involves the central area, the affected subject may complain of visual impairment which is usually bilateral and may sometimes be the presenting symptom. Branch retinal artery occusion may be extensive or limited and FA is the main imaging technique to show it; after resolution silver streaks are noted which replace the occluded arterioles. Cerebral and visual symptoms are joined to some hearing loss forms.^83^

Differential diagnoses include MS and ADEM (acute disseminated encephalo-myelitis). The differentiating feature on MRI is that the lesions related to the two latter conditions are located inside the undersurface of the corpus callosum and not in the central area as in Susac’s syndrome.^84^ Furthermore; leptomeningeal involvement is very rare with MS and ADEM. ADEM frequently involves the deep grey matter.

Susac’s syndrome has a self-limiting but long and fluctuating course, ranging from one to five years with tendency towards spontaneous improvement.

Although the etiology is unknown, findings from brain biopsies suggest small vessel occlusive vasculitis caused by an autoimmune, viral-mediated or hypercoagulability disease. In this way, ocular, brain and cochlear arteriolar micro-vascular occlusion are explained.^85^

No treatment is evidence-based because the disease is very rare. However, high doses of intravenous steroids and immuno-suppressive agents are considered to be the mainstay of therapy.

## LYMPHOMAS

The eye can be involved in both systemic and diffused hemoblastomas, particularly in B small-cell non-Hodgkin lymphoma (NHL), and in a specific form of lymphoma, primary CNS lymphoma (PCNSL) which is more appropriately defined as CNS and ocular lymphoma i.e. a single neoplastic manifestation that can be localized to the CNS, the eye, or both.

PCNSL represents 4–6% of all cases of primary CNS lymphomas and 1–2% of extranodal lymphomas.^86^ Its incidence was found to be 0.43 per 100.000 in a study by Küker.^86^ The number of new cases appears to have been increasing in the past fifteen years, probably due to the increase in AIDS and organ transplantation. These immuno-deficiencies, together with congenital types, are the three main risk factors for this disease. Female patients outnumber male subjects by a ratio of 2/1.^88^ The disease is prevalent in the fifth and sixth decades of life but rare in children and young people.

According to a an older report, ophthalmic localizations precede neurological ones^89^,^90^, however more recent surveys support the opposite. The reason may be improvements in diagnostic imaging.

When ocular symptoms (vitreous cells, exudative retinal detachment and multifocal creamy deep retinal masses) occur prior to neuro-radiologic manifestations, the disease is designated primary intraocular lymphoma (PIOL). Infrequently, lymphoma is localized to the eye or the brain, probably due to diagnostic inaccuracy.

Typically, in a female patient older than fifty, chronic uveitis without a well-defined etiology which lacks classic signs of anterior segment (synechiae, pain) or retinal inflammation (vasculitis) and is unresponsive to steroids, should raise the suspicion of lymphoma. Certain infectious agents including HSV-8, EBV and toxoplasma gondii have been suggested in the pathogenesis of this condition by stimulating B lymphocytes to evolve into lymphomatous cells.^13^,^91^–^93^

The two main pathogenic hypotheses^93^ are 1) transformation of a benign inflammatory process in the eye or the CNS into a neoplasm via monoclonal B-lymphocyte proliferation and 2) metastasis of an “occult” systemic lymphoma, eradicated from other body structures by a competent immune system to the CNS and eye because of their immunologically “privileged” characteristics. The latter suspicion is supported by positron emission tomography studies, suggesting that systemic lymphoma is present in individuals who are negative on both whole body CT scan and bone marrow biopsy.^94^ Many studies^85^,^94^ confirm that PCNSL is a large B-cell lymphoma (about 95% of all the studied cases).

From an immunogenetic point of view, the majority (60–75%) of PCNSL cases demonstrate a deletion^95^ on the long arm of chromosome 6 which contains a lymphoma-suppressor gene, perhaps the PTPRK gene involved in the regulation of lymphocyte contact and adherence^94^. This deletion does not occur in systemic lymphomas and is useful to determine the prognosis, which is more unfavourable in PCNSL.

In cerebral PCNSL, the lesions are usually supratentorial, multicentric and localized in the basal ganglia, corpus callosum and periventricular areas with a predilection for the frontal lobe.^90^ The variety of localizations corresponds to the range of neurological symptoms. Since encephalic lesions are deep-seated, epilepsy is relatively rare in contrast to other cerebral malignancies.^89^,^96^

The prevalence of frontal lobe involvement corresponds to personality and sleep disorders^86^,^89^,^96^. Age and severity of functional damage are major prognostic criteria. Meningeal symptoms indicate an even more unfavourable prognosis.

Ocular localization is rare but is the most common malignancy causing a “masquerade” syndrome. PIOL is a diffuse large B-cell lymphoma^97^ presenting with floaters, photopsia and blurred vision. It usually is unilateral at onset but becomes bilateral within one year. The typical finding is a peculiar infiltrative but not inflammatory vitritis. Lymphomatous keratic precipitates may also be seen on the corneal endothelium. FA shows RPE abnormalities, such as round hypo- or hyperfluorescent defects, because PIOL cells lodge between the RPE and Bruch’s membrane, and clumps of them can be detected by OCT imaging. Vitreous or retinal biopsy can be useful in suspicious cases for histopathological and immunohistochemical characterization.

Another very useful diagnostic tool is the detection of interleukin-10 (IL-10) in the serum and aqueous humor produced by malignant B-cells^98^; IL-10 belongs to the Th_2_ cytokine group and acts as a “growth factor” for B-cells and therefore contributes to the development and proliferation of PCNSL by inhibiting Th_1_ cytokines and consequently, T cell cytotoxic effects against malignant B-cells. Furthermore, it increases BCL-2 gene expression on B-cells and prevents their apoptosis. IL-10 is increased in PCNSL serum (similar to AIDS-associated lymphomas and EBV-related Hodgkin’s disease) and CSF. In the aqueous humor, an increased value (more than 1.0) of IL-10 and IL-10/IL-6 (a cytokine mainly produced by inflammatory cells) ratio is diagnostic for the presence of PIOL/PSNCL. In the serum, this ratio is related to both the clinical expression and the prognosis of the disease.

**Figure 14:**
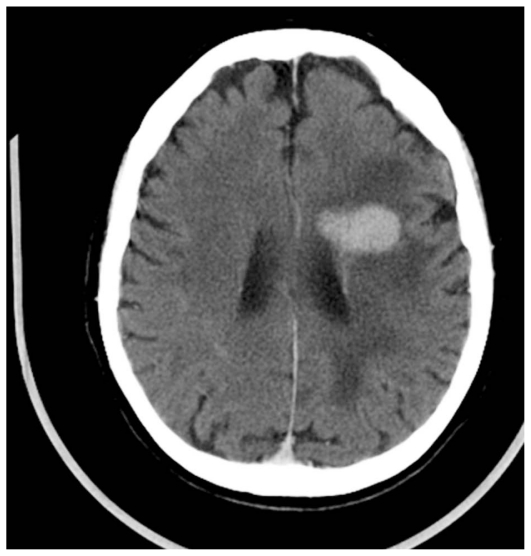
PCNSL: isolated lesion in the left brain hemisphere (homogeneous contrast enhancement on CT-scan).

Eye and brain CT are usually sufficient to make a diagnosis of PCNSL but a specific CT or MRI pattern does not exist. Some features are frequently present^89^: a) CT-scans without contrast show isodense lesions which are more common (84%) than hyperdense lesions which only account for 16%; b) contrast enhanced CT-scans show persisting homogeneous (94%), well-defined (83%) areas of contrast enhancement (Fig. 14).

Typically, lesions involve the corpus callosum, basal nuclei, or periventricular white matter^91^; they are spontaneously isodense (rarely hyperdense) and present with homogeneous contrast enhancement which is never annular, a feature in contrast to other tumor metastases. The “phantom tumor” is a name given to the “classic” disappearance of lesions after steroid therapy.

MRI without contrast shows PCNSL localizations as T_1_ hypointense and T_2_ hyperintense patterns, similar to MS.^91^ MRI with contrast shows intense well-defined, rarely annular, areas of contrast enhancement (as on CT scan).^96^ A single localization can be mistaken for a stroke.

## PARANEOPLASTIC SYNDROME

Paraneoplastic optic neuropathy (PON), has been described in cases of microcytic lung carcinoma and thymoma^99^, and presents with typical ocular symptoms, optic neuritis, retinitis, inflammatory vitritis and progressive loss of vision. Neurological signs are nonfocal encephalomyelopathy (with an inflammatory CSF), ophthalmoplegia and subacute cerebellar syndrome.^100^ This disease is caused by the presence of anti-CRMP-5 (collapsine responsive mediator protein–5) protein, which belongs to the semaphorine group i.e. proteins that play a role in the development of neuronal network in the CNS.

## CONCLUSION

As highlighted in this review, a number of disorders with heterogeneous origins present with oculocerebral symptoms and signs, with onset sometimes in one or both organs. These syndromes may be difficult to manage because they are uncommon and difficult to diagnose. Treatment requires qualified interdisciplinary knowledge and management at specialized referral centers.

## Figures and Tables

**Figure 1A:**
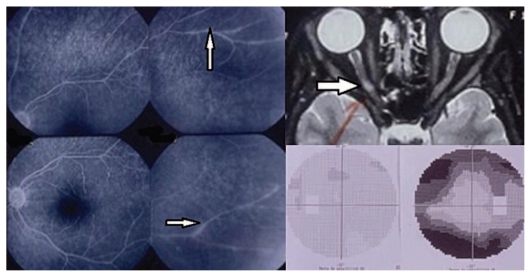
Monosymptomatic optic neuritis (MSON). Top right shows an MRI image of a demyelinating plaque of the right optic nerve with corresponding visual field changes (bottom right). Retinal vasculitis is well evident on fluorescein angiography (left frames, arrows).

**Figure 1B:**
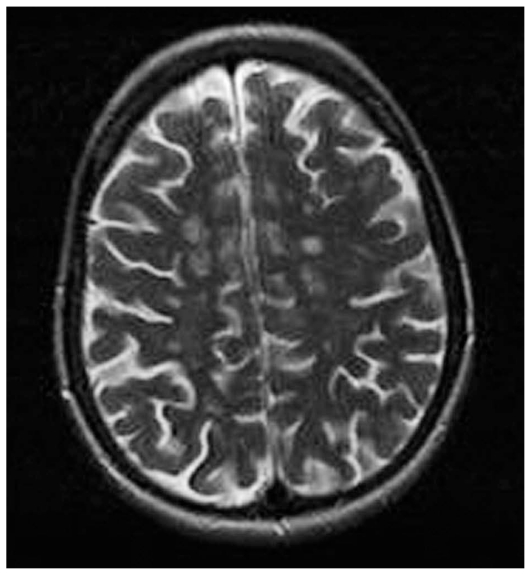
Brain MRI in multiple sclerosis shows multiple lesions (hyper-intense on T2-weighted images) located in the white matter of both hemispheres.

**Figure 2 (A–B):**
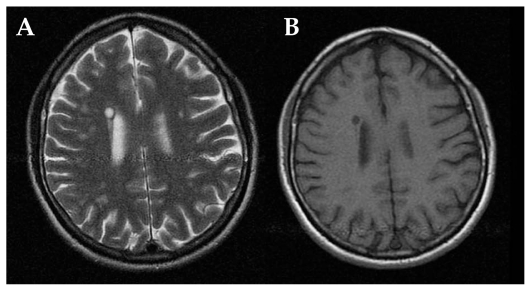
MRI in multiple sclerosis: a hyperintense parietal white matter lesion on T2 (A), appears hypointense on T1 images (B).

**Figure 2C:**
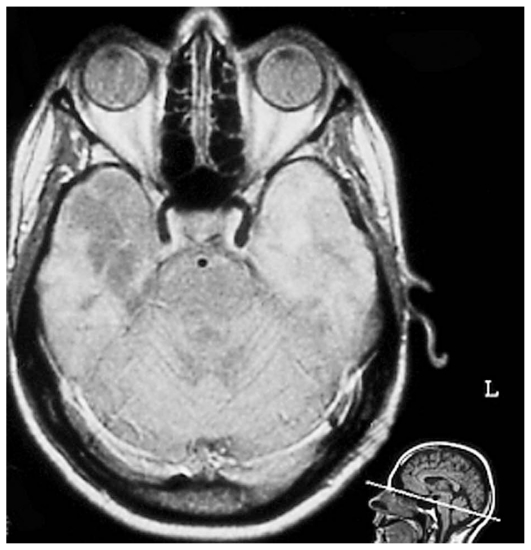
Herpes encephalitis, MRI section shows bilateral hyperdense lesions in the temporal and parietal lobes.

**Figure 5 (A and B):**
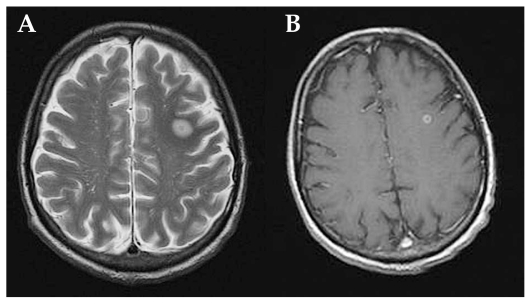
AIDS-related neuro-toxoplasmosis: single toxoplasmic lesion in the left frontal lobe (T2-weighted sequence, A), which shows ring-shaped enhancement after contrast injection (T1-weighted sequence, B).

**Figure 5C:**
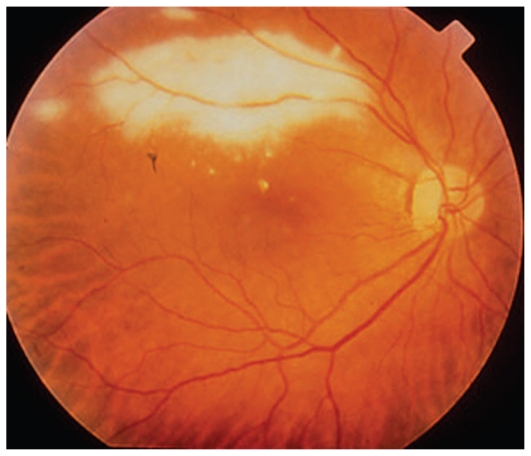
Toxoplasmic retinitis in a patient with AIDS, note the absence of inflammatory reaction.
